# Cystadénome hépatique simulant un kyste hydatique

**DOI:** 10.11604/pamj.2015.20.294.6644

**Published:** 2015-03-25

**Authors:** Mahmoudi Ammar, Faouzi Noomen

**Affiliations:** 1 Service de Chirurgie Générale et Digestive, CHU Fattouma Bourguiba de Monastir, Tunisie

**Keywords:** Foie, cystadénome hépatobiliaire, scanner, IRM, Chirurgie hépatique, liver, hepatobiliary cystadenoma, scanner, MRI, liver surgery

## Image en medicine

**Figure 1:**
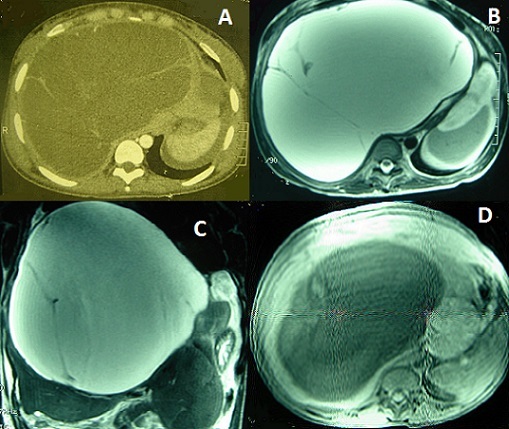
(A): TDM abdominale en coupe axiale montrant une énorme masse kystique multiloculaire de 25X13 cm occupant tout le foie droit avec des cloisons épaisses de 3 à 5 mm; (B): IRM abdominale en coupe axiale en pondération T2 montrant une masse kystique cloisonnée bien limitée du foie droit mesurant 23X23X21 cm en hyper signal homogène; (C): IRM abdominale en coupe frontale en pondération T2 montrant une masse kystique cloisonnée bien limitée du foie droit mesurant 23X23X21 cm en hypersignal homogène; (D): IRM abdominale en coupe axiale en pondération T1 montrant une masse kystique cloisonnée bien limitée du foie droit mesurant 23X23X21 cm en hyposignal

Les cystadénomes hépatiques sont des tumeurs kystiques rares et de diagnostic pré-opératoire difficile en imagerie. Ils touchent le plus souvent des femmes de plus de 50 ans. Une attitude chirurgicale radicale, compte tenu du potentiel malin de ces lésions, est recommandée. Nous rapportons le cas d'une patiente âgée de 22 ans qui a été opérée il y a 4 mois dans un hôpital régional à tort pour kyste hydatique du foie, mais en peropératoire vu l'aspect, il n'a pas été réalisé de geste. La biologie hépatique était normale. La tomodensitométrie abdominale avait objectivé une masse kystique multiloculaire de 25X13 cm avec des cloisons épaisses de 3 à 5 mm de diamètre évoquant un kyste hydatique de type III ou un cystadénome biliaire. L'IRM abdominale a objectivé une masse kystique cloisonnée bien limitée du foie droit mesurant 23X23X21 cm en hypersignal homogène T2, hyposignal T1 avec des cloisons non rehaussées après injection de Gadolinium, aspect en faveur d'un cystadénome hépatique. La sérologie hydatique était négative. Il a été réalisé dans un premier temps une ligature portale droite à fin d'entrainer une hypertrophie compensatrice du foie gauche en vue d'une résection hépatique droite. Lors de la deuxième opération, il a été réalisé une kystectomie subtotale, avec coagulation de la partie résiduelle, en raison des adhérences entre le kyste et les structures vasculaires cavo-sushépatiques. L'examen anatomopathologique avait confirmé le diagnostic de cystadénome hépatique. Les suites opératoires étaien simples et avec un recul de deux ans, il n'a pas été noté de récidive. Figure 1

